# Specific Detection of *Yersinia pestis* Based on Receptor Binding Proteins of Phages

**DOI:** 10.3390/pathogens9080611

**Published:** 2020-07-27

**Authors:** Friederike Born, Peter Braun, Holger C. Scholz, Gregor Grass

**Affiliations:** Deptartment of Bacteriology and Toxinology, Bundeswehr Institute of Microbiology (IMB), 80937 Munich, Germany; friederikeleonieborn@bundeswehr.org (F.B.); peter3braun@bundeswehr.org (P.B.); holger1scholz@bundeswehr.org (H.C.S.)

**Keywords:** *Yersinia pestis*, plague, microscopic assay, pathogen detection, receptor binding protein, phage, tail fiber, tailspike, capsule, fluorescent reporter

## Abstract

The highly pathogenic bacterium *Yersinia pestis* is the causative agent of plague, a notorious infectious zoonotic disease. When transmitted from person to person as pneumonic plague via droplets, *Y. pestis* is highly contagious and in most cases is fatal if left untreated. Thus, when plague is suspected, rapid diagnosis is crucial, as a serious course of the infection is only averted by early antibiotic therapy. The bacterium is easy to cultivate, accessible and has a high potential for nefarious use such as bioterrorism. Highly specific, rapid and easy-to-use confirmatory diagnostic methods are required to reliably identify the pathogen independently from PCR-based methods or F1 antigen-based immunological detection. *Yersinia pestis* specific phages such as L-413C and ΦA1122 are already used for detection of *Y. pestis* in bacterial plaque or biosensor assays. Here, we made use of the host specificities conferred by phage receptor binding (or tail fiber/spike) proteins (RBP) for developing a specific, fast and simple fluorescence-microscopy-based detection method for *Y. pestis*. Genes of putative RBP of phages L-413C (*gpH*) and ΦA1122 (*gp17*) were fused with those of fluorescent proteins and recombinant receptor-reporter fusion proteins were produced heterologously in *Escherichia coli*. When first tested on attenuated *Y. pestis* strain EV76, RBP-reporters bound to the bacterial cell surface. This assay could be completed within a few minutes using live or formaldehyde-inactivated cells. Specificity tests using cultures of closely related *Yersinia* species and several inactivated fully virulent *Y. pestis* strains exhibited high specificities of the RBP-reporters against *Y. pestis*. The L-413C RBP proved to be especially specific, as it only detected *Y. pestis* at all temperatures tested, whereas the RBP of ΦA1122 also bound to *Y. pseudotuberculosis* strains at 37 °C (but not at 28, 20 or 6 °C). Finally, the *Y. pestis*-specific capsule, produced when grown at 37 °C, significantly reduced binding of phage ΦA1122 RBP, whereas the capsule only slightly diminished binding of L-413C RBP.

## 1. Introduction

Plague is a notorious, highly contagious bacterial vector-borne disease typically transmitted between small mammals (rodents) and their fleas. The bacterium *Yersinia pestis*, the causative zoonotic agent of plague, is also highly pathogenic for humans. Broadly geographically distributed epidemics of the plaque bacterium had major social impact and economic consequences in the preantibiotics era. *Y. pestis* has caused three historically well-documented severe plague pandemics in the mid-6th, the mid-14th and the early 20th century [1].

The high pathogenicity of *Y. pestis* is largely due to its unique ability to successfully counter the defense mechanisms of both mammals and insects and to adapt to temperature fluctuations between 0 and 37 °C during its natural life cycle [2]. This includes the adjustment from a cold-blooded flea vector (20–28 °C) to a warm-blooded mammalian host (37 °C) and infected animals during hibernation (6 °C). This adaptation to different growth temperatures has been associated with changes in the surface structure of *Y. pestis* [3].

During infection of mammals, *Y. pestis* secretes a surface antigen called fraction 1 (F1) protein forming a gel-like capsule [4]. This capsule is an important protective antigen for *Y. pestis* [5]. Besides being strongly antigenic, the capsule of *Y. pestis* functions as a virulence-associated surface structure that has antiphagocytic activity reducing uptake by macrophages and epithelial cells. *Y. pestis* is the only *Yersinia* species that at temperatures >30 °C produces this capsule composed of Caf1 subunits encoded by the *caf1* gene located on plasmid pFra [5,6].

The genus *Yersinia* contains two well-known mammalian enteropathogens, both of which cause yersiniosis, *Y. enterocolitica* and *Y. pseudotuberculosis*, the latter being closely related to *Y. pestis*. With the availability of genome sequences, a variety of *Yersinia* isolates originally typed as *Y. pseudotuberculosis* have now been reclassified into other species. These bacteria now belong to a group called “*Y. pseudotuberculosis* complex” including *Y. pestis*, *Y. pseudotuberculosis*, *Y. similis* and *Y. wautersii* [7].

After suspected infection with *Y. pestis*, rapid diagnosis is required, since a fatal course of the disease can only be averted by prompt antibiotic therapy. Antigen diagnostic methods (e.g., Enzyme-linked Immunosorbent Assay, ELISA), in which antigen detection is depending on antibodies, are very reliable [8,9,10], however, the assays are time consuming and have the disadvantage of delayed detection, since positive results can only be reliably established several days after the bacterium has multiplied considerably inside the host. Therefore, these methods are typically used for confirmation only. Currently, the detection of *Y. pestis* is generally founded on a combination of several methods. Culture-based identification by subsequent microscopy provides rather tentative results; more precise results are obtained by the detection of antigens (e.g., the F1 capsule antigen) and by molecular genetic identification using polymerase chain reaction (PCR) e.g., on virulence gene markers *pla* and *caf* constituting a diagnostic standard for the plague pathogen [11,12]. PCR can also be combined with other means of detection such as bacteriophage (phage) plaque diagnostics but such methods take considerable time [13]. The time issue can be ameliorated when recombinant, light emitting phages are constructed for assays measured in hours instead of days [14]. A clear advantage of additional rapid and specific microscopy-based detection methods would be that these could independently confirm PCR results: a combination necessary for a complete diagnostic algorithm.

We hypothesized that receptor binding proteins (RBP) of host-specific bacteriophages of *Y. pestis* [15,16] could close this diagnostic gap. Phages are the most numerous and versatile organisms on earth [17]. For *Y. pestis* phages, as for other phages, receptor recognition is usually a highly specific process and is part of the natural mechanism of host adsorption [18,19]. The susceptibility of a bacterium to phage infection primarily depends on whether the phage is able to find its specific attachment sites, i.e., its receptors on the host cell surface. Phages can be divided into temperate or lytic phages based on their life cycles. In contrast to temperate phage, infection by lytic phage always leads to lysis of the bacterial cell and to the release of new phage progeny. However, the first step of an infection cycle is always the adsorption of the phage on the bacterial surface. In tailed phages, this binding is usually mediated by phage structural proteins, the RBP [20].

The order Caudovirales (tailed phage) consists of the three families: *Myoviridae*, *Siphoviridae* and *Podoviridae* and comprises about 96% of all known phages [20]. The phages specific to the genus *Yersinia*, the *Yersinia* phages, also cover these families. Among these, *Y. pestis* phages differ in their antigenic properties, morphology of their virions, host virulence, genome structure and degree of specificity for the pathogen. Two well-characterized diagnostic phages of *Y. pestis* (relevant for this work) are L-413C [15] and ΦA1122 [16]. Phage ΦA1122 belongs to the *Podoviridae* family and has a short noncontractile tail. Sequence analyses showed strong similarity of the ΦA1122 phage to the *Escherichia coli* phages T3 and T7 [16]. Phage L-413 was isolated from the *Y. pestis* strain 413 and after several passages in the *Y. pestis* host strain, the lytic mutant L-413C phage was obtained, which only lyses *Y. pestis* but not *Y. pseudotuberculosis* or the vast majority of *E. coli* strains [15,21]. Phage L-413C is similar to the P1, P2 and P4 phages [15] belonging to the *Myoviridae* family featuring a relatively long contractile tail. Previously, it has been shown that the plague diagnostic phages L-413C and ΦA1122 are very specific to *Y. pestis* and allow the differentiation of *Y. pestis* from *Y. pseudotuberculosis* strains [15,16].

For asserting host specificity and to initiate the infection process, diverse phage tail structures constitute key factors of tailed phages. RBP at the distal end of these tails interact with bacterial cell surface structures. Tail fiber, tailspike or tail tip (or tail nozzle) proteins can act as RBP and specifically recognize host receptors such as cell wall lipopolysaccharides (LPS), teichoic acids or proteins (e.g., porins) [20]. Typically, RBP are homotrimeric complexes and often consist of two critical domains. The amino terminus of the RBP typically connects the RBP to the rest of the phage, whereas the carboxy terminus, which is oriented away from the phage, forms the binding domain for the host receptor [22,23,24]).

Receptor recognition of phages of the T7 group is mediated by the tail fiber protein Gp17. Due to the similarity of the ΦA1122 phage to other phages of the T7 group, Garcia et al. (2003) and Kiljunen et al. (2011) postulated that Gp17 of ΦA1122 was responsible for receptor recognition [16,18]. The RBP of the T7 and T3 phages are 98.9 and 81% identical to Gp17 of ΦA1122, respectively. It was therefore assumed that ΦA1122 can as well bind to LPS. Protein GpH of the L-413C phage is the likely tail fiber protein of this phage [15,19]. For GpH a possible function as a RBP was inferred from similarity to its homolog in P2-like phages [15]. Since the host range of P2-like phages is determined by the tail fibers, the authors postulated host specificity is exerted by GpH, the closely related putative tail fiber protein of the phage L-413C.

From this knowledge base we aimed to experimentally test the hypotheses put forth earlier through in silico considerations [15,16] that the putative gene products of *gp17* (phage ΦA1122) and *gpH* (phage L-413C) constitute the RBP of these *Y. pestis* phages. For this, the putative phage RBP of phages L-413C and ΦA1122 were produced in heterologous host *E. coli* coupled to fluorescent reporter proteins and the recombinant proteins purified. From there we developed a specific fluorescence microscopy detection assay for *Y. pestis* using these RBP fusion reporter proteins by testing specificities to cells of *Y. pestis* and other *Yersinia* spp. under different growth temperatures and capsule inducing conditions.

## 2. Results

### 2.1. Fluorescent Fusion Proteins of the Putative RBP of Phages L-413C or ΦA1122 Can be Heterologously Produced in E. coli

The putative RBP genes of the *Yersinia* phages L-413C (*gpH*) and ΦA1122 (*gp17*) were cloned as fusions with genes of fluorescence reporter proteins (eGFP and mCherry) and a Twin-Strep-tag (TST) epitope (located at the N-terminus of the resultant fusion protein). Recombinant expression plasmids tested positive by colony PCR and Sanger DNA-sequencing were transferred into production strain *E. coli* ArcticExpress and protein biosynthesis was initiated. Cells were lysed and cleared lysates used for affinity chromatography. Expected sizes of fusion proteins were confirmed by Western blot analysis. Supplementary Figure S1 shows representative stained bands of eGFP-RBP (phage L-413C GpH; 130 kDa) and eGFP-RBP (phage ΦA1122 Gp17; 93.5 kDa) fusion proteins.

### 2.2. Binding of Fluorescent RBP Fusion Proteins of Phages L-413C and ΦA1122 to *Y. pestis* Cells

**Figure 1:**
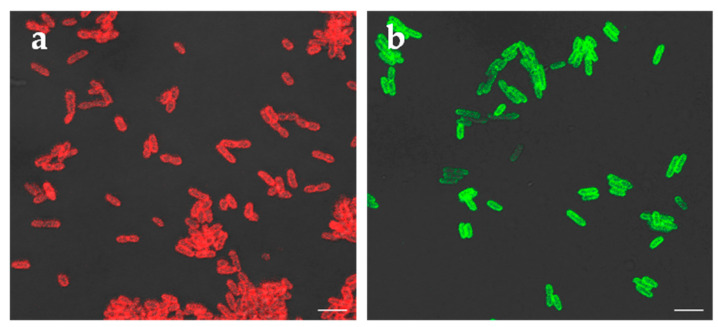
Binding of fluorescent receptor binding proteins (RBP) fusion reporters of *Yersinia* phages L-413C and ΦA1122 to *Y. pestis* cells. (**a**) mCherry fused to RBP of phage L-413C (red signals) or (**b**) eGFP fused to RBP of phage ΦA1122 (green signals) were added to cells of logarithmically growing *Y. pestis* cultures, washed with buffer to remove unbound reporter proteins, transferred to microscopy slides and subjected to fluorescence microscopy. Shown are representative fluorescence signals recorded at 630× magnification (scale bar: 5 µm).

Next, the putative RBPs were examined for binding to *Y. pestis* cells. For this, a culture of *Y. pestis* strain EV76 was grown to exponential (logarithmic) growth phase at 28 °C and samples processed for fluorescence microscopy. For analysis of binding of phage L-413C RBP, a red fluorescence reporter (mCherry) and for phage ΦA1122 RBP a green fusion reporter (eGFP) was used. The results depicted in Figure 1 strongly supported the hypothesis predicting that GpH is the RBP of phage L-413C and Gp17 that of phage ΦA1122. Each of the two RBP fusion reporters extensively attached to the surfaces of *Y. pestis* cells. This test was fast; the steps from harvesting cultures to imaging and taking pictures can be accomplished within approximately 20 min.

### 2.3. Time Course of Fluorescent RBP Fusion Protein Binding to *Y. pestis* Cells

For the next line of experiments, RBP binding at several different culture temperature regimens was tested. This was done to address the temperature-dependent structural changes of the LPS composition of *Y. pestis*. Different pairs of adjacent, inner nuclear residues of the LPS, more precisely the terminal residue of the outer core (N-acetylglucosamine), were previously identified as surface receptors for phage L-413C and 3-deoxy-D-manno-octulosonic acid (Kdo) and its isosteric 3-hydroxy derivative d-glycero-α-d-talo-oct-2-ulosonic acid (Ko) for phage ΦA1122 [18,19]. In order to mimic different hosts of *Y. pestis*, the growth temperature of *Y. pestis* cultures were varied and binding of the RBP-reporters analyzed. Individually, the different growth temperatures were to represent the typical body temperature of an active mammal (37 °C), the optimum growth temperature of *Y. pestis* (28 °C), the (ambient) temperature in flea host (20 °C) and the body temperatures of a mammal in hibernation (6 °C). For each growth temperature, culture samples were taken hourly for a duration of 12 h and after 24 h in order to record temperature-dependent time-courses of RBP-reporter binding.

**Figure 2:**
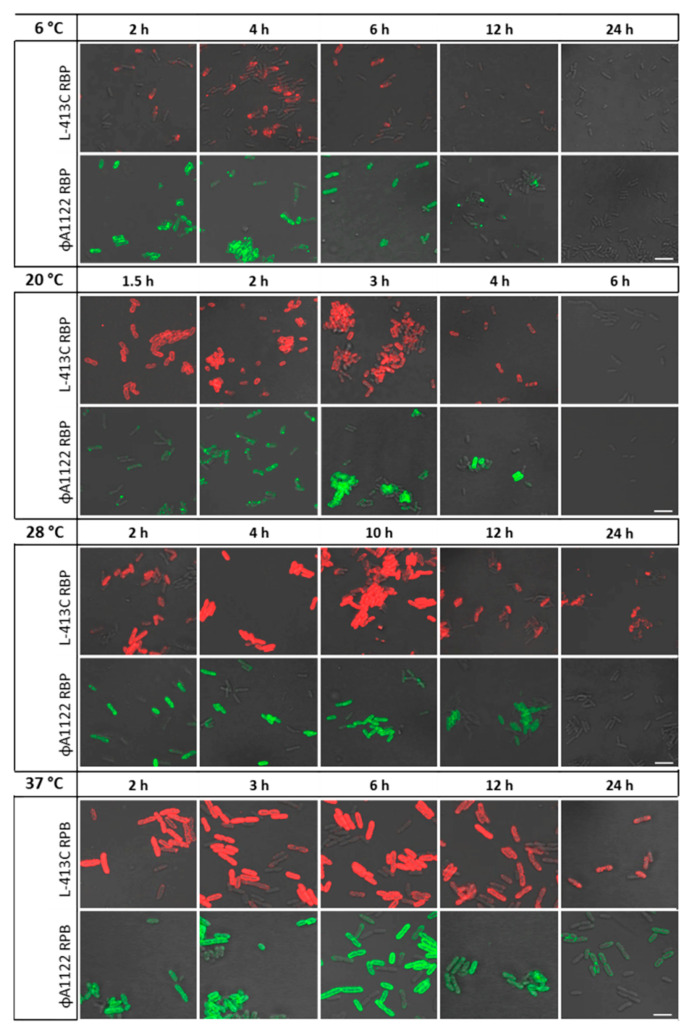
Time course of binding of *Yersinia* phage RBP fusion reporters to *Y. pestis* cells. Cultures of *Y. pestis* EV76 were grown from lag to exponential (logarithmic) and stationary phase and samples withdrawn at representative time points for RBP binding assays. Individual micrographs are shown for mCherry-RBP fusion protein (phage L-413C; red signal) or eGFP-RBP fusion protein (phage ΦA1122; green signal) binding to *Y. pestis* cells at 6, 20, 28 and 37 °C (recorded in merged light and fluorescence channels; scale bar: 5 µm).

Figure 2 shows binding of the mCherry-RBP (phage L-413C) or the eGFP-RBP (phage ΦA1122) fusion proteins at representative time points during growth at temperatures listed above. Already at early logarithmic phases after 2 h, RBP binding was observed across all temperatures tested. With the exception of the 6°C culture, fluorescence (red) mediated by binding of phage L413C-RBP to bacterial cells appeared to be more extensive than that of phage ΦA1122-RBP. Fluorescence increased over the next hours and strong signals were recorded for phage L413C-RBP binding after 4 h (6 °C), 2–3 h (20 °C), 4–10 h (28 °C) and 2–12 h (37 °C) as well as of phage ΦA1122-RBP binding after 2–6 h (6 °C), 3–4 h (20 °C), 2–10 h (28 °C) and 2–6 h (37 °C). Overnight (24 h) cultures only fluoresced dimly (both RBP at 37 °C) or patchy (phage L413C-RBP at 28 °C) or not at all (both RBP-reporters at 6 and 20 °C as well as phage ΦA1122-RBP at 28 °C).

**Figure 3:**
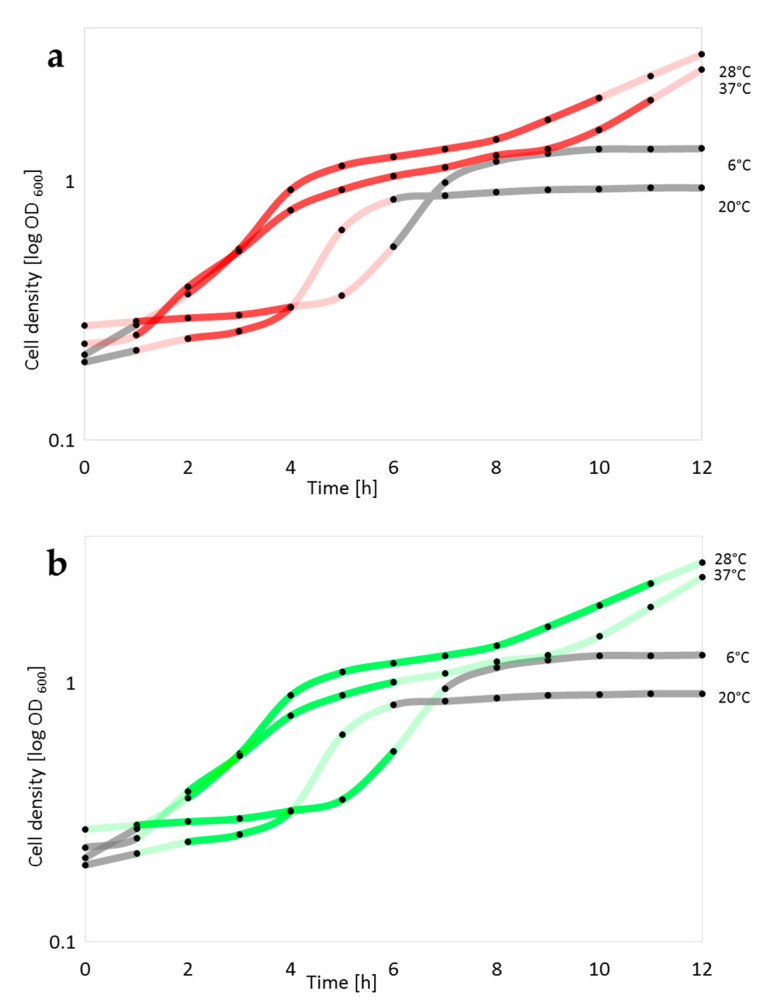
Semiquantitative representation of binding of *Yersinia* phage RBP fusion reporters to *Y. pestis* cells. Micrographs taken from culture samples withdrawn at different time points (growth phases) from growth experiments at 6, 20, 28 or 37 °C (see Figure 2 for representative examples) were visually analyzed for RBP-reporter binding to *Y. pestis* EV76 cells. (**a**) mCherry-RBP (phage L-413C; red) or (**b**) eGFP-RBP (phage ΦA1122; green) fusion proteins. Binding of the reporters was assessed by an increase or decrease (light colors) of fluorescence signals (and phases with constant strong signals; dark colors) and plotted onto data (black dots) from actual growth curve experiments (measurements of optical culture densities at 600 nm). Periods with lack of visual fluorescence signals indicating no RBP binding, were scored as grey lines.

Binding of the RBP-reporter proteins was also assessed in a more semiquantitative manner (by visual comparison) as the increase (or strength) of the fluorescence signal over time, which indicated increased (extensive) binding of the fusion proteins, or as diminished fluorescence signal, which indicated diminished binding of the fusion proteins (Figure 3). For this, analysis of RBP-reporter binding was coupled to culture growth experiments from lag to logarithmic and stationary growth phases. In Figure 3 grey lines indicate periods of cell growth in which the RBP-reporters did not bind at all. During periods of extensive RBP-reporter binding, cells fluoresced strongly (indicated as dark-colored lines), while for transition phases, diminished fluorescence signals are represented by light colors.

For binding of the mCherry-RBP fusion protein (phage L-413C) to *Y. pestis* EV76 at 6 °C, after weak binding right on from the beginning, further increase of the fluorescence signal was detected from 1 h onwards, which indicated extensive RBP binding during this time period (Figure 3a). A decrease in fluorescence signal intensity, indicating diminished RBP-reporter binding, was detected after 4 h but the signal could be detected until 6 h. Thus, the optimum time period for binding of this RBP at 6 °C was late lag and early exponential phase of the cells in culture. At 20 °C the signal strength increased after 1 h. The range with constant strong signal strength was 2 to 4 h, followed by a decrease of the fluorescence signal over the next 2 h. An extended binding period was observed for RBP binding at 28 °C. The maximum fluorescence period ranged from 2 to 10 h. The longest maximum intensity binding period of phage L-413C-RBP was recorded from cells cultivated at 37 °C. This period lasted from 1 to 11 h and then the signal declined. 

Similar results were obtained for binding of the eGFP-RBP (phage ΦA1122) fusion protein (Figure 3B). At 6 °C, strong fluorescence signals were observed from 1 to 6 h, at 20 °C a short maximum binding period was reached between 2 and 4 h after culture inoculation. At 28 °C the longest maximum binding period for this RBP was recorded, lasting from 2 to 11 h. Finally, at 37 °C this period diminished to 2 to 6 h fading out with completion of exponential phase growth. 

Notably, binding of the mCherry-RBP (phage L-413C) fusion protein to cells produced a significantly weaker overall fluorescence signal at 6°C than at 20–37 °C. This was also observed for the eGFP-RBP (phage ΦA1122) fusion protein: weaker fluorescence signal at 6 °C, but the strongest signals for this RBP fusion were observed at 28 °C and 37 °C (compare Figure 2). We also noted that *Y. pestis* EV76 cells became significantly larger at an incubation temperature of 37 °C than at all other temperatures (including the organism’s preferred growth temperature of 28 °C; compare Figure 2).

Taken together, the proposed function of GpH (of phage L-413C) and Gp17 (of phage ΦA1122) by employing fluorescent fusion protein technology could be experimentally supported. Thus, GpH and Gp17 are most likely the RBP of their respective *Yersinia* phages and these RBPs bind to their host cells at several relevant growth temperatures.

### 2.4. The *Y. pestis* Capsule does not Seem to Impair Phage L-413C RBP Binding to Cells Whereas Binding RBP of Phage ΦA1122 is Diminished

**Figure 4:**
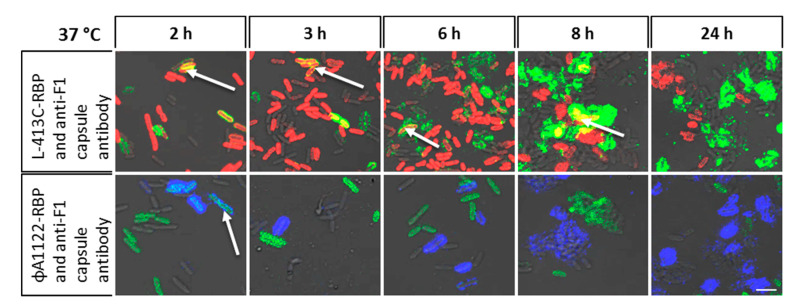
Binding of RBP fusion reporters to growing cultures of *Y. pestis* EV76 cells at 37 °C. In this representation, capsule formation is detected by means of monoclonal anti-F1 capsule antigen antibody in combination with secondary antibody labelled with Alexa Fluor 488 (green fluorescence, upper panels only) for co-detection with L-413C-RBP or Alexa Fluor 647 (false color blue fluorescence, lower panels only) for co-detection with ΦA1122-RPB, respectively. RBP binding to *Y. pestis* EV76 cells at indicated time points at 37 °C is shown as individual representative micrographs for phage L-413C mCherry-RBP-reporter (red signals) or phage ΦA1122 eGFP-RBP-reporter (green signals) as merges with fluorescent antibody signals (and recorded in merged light and fluorescence channels). Arrows indicate doubly labelled cells with RBP-reporters and antibodies (scale bar: 5 µm).

The capsule-like fraction 1 (F1) antigen expressed by *Y. pestis* is a well-characterized, specific marker for the identification of the plague pathogen. Capsule biosynthesis by *Y. pestis* occurs at cultivation conditions above 30 °C. [5,6]. This physical proteinous barrier can be expected to have a negative influence on the binding of RBPs. To test this, detection of *Y. pestis* by binding of RBP-reporters was combined with antibody-mediated detection of the F1 antigen. Figure 4 shows binding of the fusion reporters mCherry-RBP (phage L-413C) and eGFP-RBP (phage ΦA1122), respectively, to growing *Y. pestis* cultures during a time period from 0 and 24 h after inoculation. Capsule formation was visualized by monoclonal antibodies against the F1 capsule antigen coupled with a secondary fluorescent detection antibody. The fluorescence signal of the antibody increased over time, indicating capsule formation (Figure 4). Concomitantly, the fluorescence signal of the ΦA1122-RBP-reporter decreased, suggesting diminished RBP binding in the presence of capsule. Of note, only a fraction of cells synthesized capsules and this fraction increased over time. Thus, RBP-reporter binding to non-encapsulated cells was still possible.

Binding of fusion protein mCherry-RBP (phage L-413C) yielded different results. Despite increasing fluorescence signals caused by anticapsule antibody binding to cell surfaces, no marked decrease in the fluorescence signal of the RBP-reporter was noted over the covered time period (Figure 4). Even when the fluorescence signal of the antibody was strong, cells showing superimposition of the fluorescence signals of both the antibody and the RBP-reporter were visible (yellow cells labelled by a blue arrow in Figure 4; upper 8 h micrograph). This indicated largely unhindered RBP binding to cells despite capsule formation. Thus, capsule formation had only a negligible influence on the RPB protein of phage L-413C. Similar to the results for phage ΦA1122 RBP, not all cells were capsulated, so all cells were labelled by the phage L-413C-RBP-reporter (compare also RBP binding in Figure 2, 37 °C micrographs of cells without capsule staining). Cells grown at 28 °C did not produce any capsule and thus were not labeled by anticapsule F1-antigen antibodies (Figure S2). Capsule formation detected by anti-F1 antigen antibody staining of cells grown at 37 °C (Figure 4) was confirmed by using the Plague Bio Threat Alert rapid test performed with the same *Y. pestis* EV76 cultures used for microscopy. However, in contrast to the RBP-based assay, the lateral flow assay solely recognizes the presence of capsulated cells but not their relative abundance.

### 2.5. Binding of Phage RBP-Reporters is Specific for *Y. pestis* Cells

**Figure 5:**
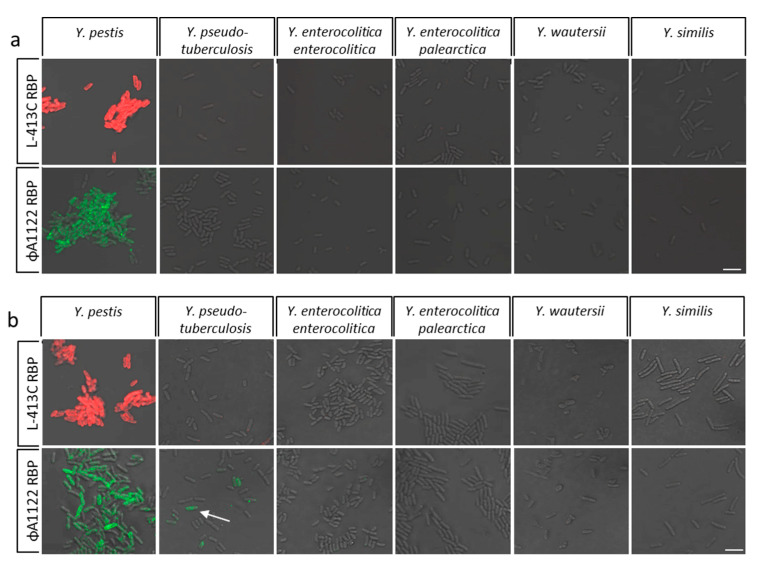
Specificity tests of the RBP-reporters binding to cells of *Yersinia* spp. closely related to *Y. pestis*. Cultures of several *Yersinia* spp. (as indicated) and of *Y. pestis* EV76 as positive control were grown at 28 °C (**a**), or 37 °C (**b**). After 4 h of growth, samples were withdrawn, incubated with mCherry-RBP-reporter (from phage L-413C; red signals) or eGFP-RBP-reporter (from phage ΦA1122; green signals) and subjected to fluorescence microscopy (recorded in merged light and fluorescence channels). The arrow indicates an individual cell labelled by the RBP-reporter (scale bar: 5 µm).

In the next series of experiments, we examined RBP binding to cells of species closely related to *Y. pestis*. These comprised risk group 2 pathogens of the genus *Yersinia* such as *Y. pseudotuberculosis*, *Y. enterocolitica* subsp. *enterocolitica* and *Y. enterocolitica* subsp. *palearctica*, *Y. wautersii* and *Y. similis*. Binding tests were conducted with bacterial cultures grown at 28 and 37 °C, respectively. Figure 5 shows specificity tests for mCherry-RBP (phage L-413C, red signals) and eGFP-RBP (phage ΦA1122, green signals) as representative micrographs after 4 h of bacterial growth, a time period optimum for RBP binding (compare Figure 3). RBP binding at additional cultivation time points was also analyzed with similar results. At 28 °C, neither RBP-reporter bound to cells of any other *Yersinia* spp. except *Y. pestis* (Figure 5a).

When cultures were grown at 37 °C, *Y. enterocolitica*, *Y. wautersii* and *Y. similis* yielded no fluorescence signals (Figure 5b) indicating the absence of either RBP-reporter binding to these cells. In contrast, cells of *Y. pseudotuberculosis* were partially labelled by RBP of phage ΦA1122 but not by RBP of phage L-413C at this growth temperature. It can therefore be proposed that binding of either RBP to *Y. pestis* was specific to cells grown at 28 °C. However, only the RBP of phage L-413C was able to differentiate *Y. pestis* from its genetically closest relative *Y. pseudotuberculosis* at 37 °C. 

**Figure 6:**
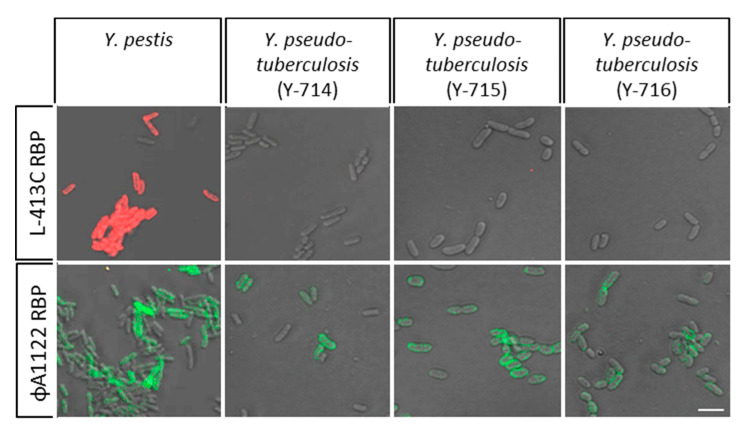
Binding of RBP-reporters to cells of additional *Y. pseudotuberculosis* strains at 37 °C. Cultures and samples of three *Y. pseudotuberculosis* O:1a strains were treated as described in Figure 5), mCherry-RBP (from phage L-413C; red signals) or eGFP-RBP fusion proteins (from phage ΦA1122; green signals) were added and samples subjected to fluorescence microscopy (recorded in merged light and fluorescence channels; scale bar: 5 µm).

To further investigate RBP binding to *Y. pseudotuberculosis* cells at 37 °C, additional isolates were tested as shown in Figure 6. The red RBP-reporter of phage L-413C yielded no fluorescence signals and thus did not bind to these cells. In contrast, binding of the green RBP-reporter of phage ΦA1122 to *Y. pseudotuberculosis* cells was confirmed. As a consequence, it seems not advisable to use the phage ΦA1122 RBP for identification of *Y. pestis* grown at 37 °C, e.g., when provided as a clinical sample from a patient. Instead, application of the RBP-reporter of phage L-413C is probably a better, more specific choice.

### 2.6. Specificity Testing of Phage RBP-Reporters on Inactivated *Y. pestis* Cells of Risk Group 3

**Figure 7:**
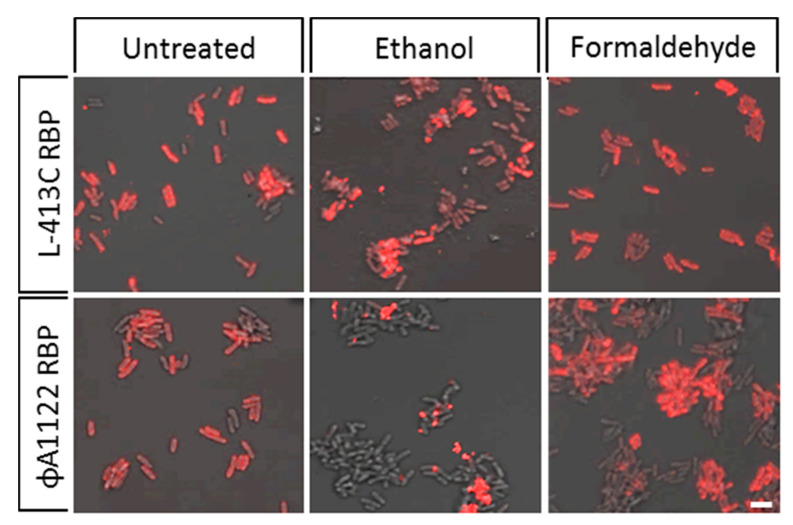
Binding of RBP-reporters to inactivated cells of *Y. pestis*. Cells of *Y. pestis* EV76 grown for 7 h at 28 °C were inactivated as indicated, washed with PBS buffer, incubated with fusion proteins mCherry-RBP (phage L-413C; red signals, upper panels) or mCherry-RBP (phage ΦA1122; red signals lower panels) and subjected to fluorescence microscopy (recorded in merged light and fluorescence channels). Untreated cells served as positive controls (scale bar: 5 µm).

Experimentation with live, highly pathogenic *Y. pestis* cells of risk group (RG) 3 is often not practical or readily possible, e.g., in field settings. To address this challenge, four different inactivation methods for *Y. pestis* were compared for their compatibility with subsequent RBP binding assays. For all methods, complete sterility of the RG-2 test organism *Y. pestis* EV76 was confirmed by cultivating the inactivated cells. We found that both inactivation with peracetic acid or with heat resulted in strong morphological changes to the dead cells and therefore the use of these methods as an upstream step prior to RBP-reporter labelling and microscopy was abandoned. Inactivation with ethanol led to the formation of cell clumps. As a consequence, subsequent RBP binding tests using cells inactivated with ethanol showed rather punctiform, non-uniform fluorescence signals that did not cover entire surfaces (Figure 7, center micrographs). Inactivation with 4% paraformaldehyde (PFA), however, proved to be the most suitable method, both in terms of conserving cell morphology, of retaining strong fluorescence signals and of uniform RBP binding to the entire cell surface (Figure 7, right micrographs). Therefore, inactivation with 4% PFA was used for subsequent specificity assays on RG-3 *Y. pestis* strains next.

**Figure 8:**
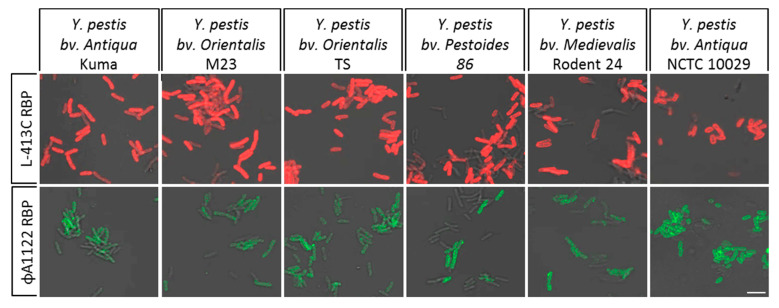
Binding of RBP-reporters to inactivated cells of RG-3 *Y. pestis*. Cells of *Y. pestis* grown for 7 h at 28 °C on solid BHI media were inactivated with 4% PFA. Inactivated cells were incubated with fusion proteins mCherry-RBP (phage L-413C; red signals, upper panels) or eGFP-RBP (phage ΦA1122; green signals lower panels) and subjected to fluorescence microscopy (recorded in merged light and fluorescence channels). Strains tested represent a phylogenetically diverse set of *Y. pestis* isolates as indicated (scale bar: 5 µm).

In order to avoid the cultivation of RG-3 *Y. pestis* strains in liquid medium in a BSL-3 environment, we tested using RG-2 *Y. pestis* EV76 whether cultivation on solid growth media is compatible with RBP binding. This test was carried out over a cultivation period of up to 24 h. Between 6 to 8 h, strong fluorescence signals could be detected indicating binding of the fusion proteins mCherry-RBP (phage L-413C) and eGFP-RBP (phage ΦA1122) to the bacterial surfaces. Next, a variety of six RG-3 *Y. pestis* strains of diverse phylogenetic subgroups were grown on solid BHI media for 6 to 7 h at 28 °C and then inactivated by PFA. After incubation with RBP-reporters, strong fluorescence signals were detected for cells of all six RG-3 *Y. pestis* strains using either reporter, mCherry-RBP (phage L-413C) or eGFP-RBP (phage ΦA1122) indicating efficient RBP binding to inactivated cells (Figure 8). This confirmed results obtained using attenuated RG 2 strain *Y. pestis* EV76 (compare Figure 7). Thus, cultivation on solid media and inactivation with 4% PFA was suitable for assaying RG-3 *Y. pestis* strains. Moreover, microscopic re-examination of the inactivated cells two weeks after inactivation could still be successfully performed. There were no changes in the cell morphologies and efficacies of RBP-reporter binding to the PFA-inactivated cells when samples were stored at 4 °C.

## 3. Discussion

In *Y. pestis*, as in most Gram-negative bacteria which are surrounded by an outer membrane, the inner layer of the outer membrane contains lipoproteins whereas the outer layer features LPS moieties which constitute a heterologous population of amphiphilic macromolecules [2]. In addition, embedded in this unique membranous structure are outer membrane proteins (OMP) which play a critical role in translocation of solutes and proteins, in pathogenesis, as well as in signal transmission. Both OMP and LPS can act as specific phage receptors [18,19,20].

The specificity of phage-host interactions is typically defined by both phage RBP and the type and structure of host receptor(s). For this, receptor localization, as well as receptors abundance and density on the cell surface may modulate specificity [20]. Phylogenetically different phages infecting the same bacterial species often have different receptors [20]. This is also observed for the two *Yersinia* phages L-413C and ΦA1122. Each of these phages binds to different structures of the *Y. pestis* LPS core. While the L-413C phage uses the terminal residue (N-acetylglucosamine) of the LPS as surface receptor, the surface receptor of phage ΦA1122 is 3-deoxy-D-manno-octulosonic acid (Kdo) and its isosteric 3-hydroxy derivative d-glycero-α-d-talo-oct-2-ulosonic acid (Ko). In addition to these critical receptor structures, two pairs of adjacent LPS sugar residues of the LPS core are also involved in phage adsorption [19].

The LPS core structures of Gram-negative bacteria contain several heptoses, as well as glucose and galactose, which are tethered to lipid A via Kdo. Only a limited diversity of LPS between species is observed [25]. The LPS of *Y. pestis* differs from that of other *Yersinia* spp. in that it is rough, i.e., the O-antigen polysaccharide chain consisting of oligosaccharide repeat units is missing [2,18,25]. The missing O-polysaccharide chain is due to a nonfunctional O-antigen gene cluster caused by several frame-shift mutations [26,27]. The LPS of *Y. pestis* therefore consists of a short carbohydrate chain bound to lipid A [18,28]). The inner nuclear region acts as a receptor for most bacteriophages specific for the LPS of *Y. pestis* but the lipid A and nuclear oligosaccharide structures of *Y. pestis* and *Y. pseudotuberculosis* LPS are largely identical [2]. The S-type LPS, which is typical for most bacteria forming smooth colonies and is also found in *Y. pseudotuberculosis* and *Y. enterocolitica* [26], additionally contains the O-antigen (oligosaccharide repeat units) [2]. In *Y. enterocolitica* and *Y. pseudotuberculosis*, O-antigen expression is temperature regulated. Bacteria cultivated at room temperature produce large amounts of O-antigen, whereas at 37 °C its production is diminished [25].

From in vitro analysis, authors of previous studies suggested that the gene products of the genes *gpH* [phage L-413C] and *gp17* [phage ΦA1122] likely represent the tail fibers RBP of these phages [15,16]. Typically for tail fibers, the N-terminus of the RBP connects the RBP with the rest of the phage, whereas the C-terminus, which is oriented away from the phage, forms the binding domain for the host receptor [22,23,24]. Nobrega et al. (2018) described that, due to the length and complexity of tail fiber proteins, chaperones are often required for their proper folding and functionality [20]. Garcia et al. (2008) proposed that the gene product of *gpG* located downstream of the sequence of the gene of proposed tail fiber protein GpH in the genome of phage L-413C encodes a protein (GpG) that serves the assembly of the tail fiber protein [15]. This notion is supported by data from the closely related P2 phage. Here, gene product G possesses a function in the assembly of the gene product H which is the tail fiber protein in P2 phage [29]. Coexpression of the *gpH* RBP gene (of phage L-413C) with the downstream *gpG* gene, however, did in our hands not improve RBP yield or solubility (unpublished results).

The present work revealed different host specificities of the RBP from phages L-413C and ΦA1122, respectively. Garcia et al. (2008) suggested the mosaic genetic origin of the L-413C-RBP (GpH) protein being responsible for its ability to distinguish between *Y. pestis* and *Y. pseudotuberculosis* cells [15]. The authors postulated a hybrid protein pattern composed of four different regions for this RBP. Two sections of the GpH protein, the N-terminal 207-(≥91% match) and C-terminal 137 amino acids (59% match), have a significant similarity with the RBP of phage P2 protein H [15]. The remaining middle part of the phage L-413C-RBP is not homologous to that of protein H from P2 but rather seems to be a mosaic itself with highest partial similarities to other enterobacterial phage tail fiber proteins. The authors proposed this GpH (the RBP) to be a hybrid resulting from recombination events. The same authors also proposed that the specific substitutions in the polypeptide were responsible for changes in the host recognition region that sculptured the particularly high specificity of the L-413C phage towards *Y. pestis* [15]. In contrast, the RBP gene of phage ΦA1122 shows an overall nucleotide sequence identity of 85.3% with the phage T7-RBP gene 17 (*gp17*) [30], whereby the first 500 nucleotides at the 5′-end are 93.6% identical, while the 500 nucleotides at the 3’-end are only 73.4% identical [16]. Similar to the RBP gene of phage L-413C, the authors concluded that an evolutionary recombination event led to a change in the phage ΦA1122 host spectrum [15]. Though the RBPs characterized in this work originate from unrelated phages, genetic rearrangements, mutations and likely horizontal gene transfer thus have achieved, each from a unique phage progenitor chassis, very high host specificities.

The capsule-like fraction 1 (F1) antigen produced by *Y. pestis* is a specific marker for pathogen identification. It is thus not surprising that the F1 antigen is the basis of a variety of detection methods [31]. F1 antigen biosynthesis is unique to *Y. pestis* and upregulated at 37 °C (mammalian phase of the infection cycle) [5,6]. It is believed that the induction of expression at 37 °C is related to the capsule’s role in protecting the bacterium from being attacked and killed by phagocytic host cells [6]. At temperatures below 37 °C, the expression level of the F1 antigen gene is significantly reduced, limiting F1 tests on cells grown under such conditions [32]. Initially, we had expected that RBP-dependent detection of *Y. pestis* as described in this work would be negatively impeded by the presence of F1 antigen at 37 °C. Because the F1 antigen is anchored to the outer membrane [6], it can be assumed that capsule subunits on the cell surface would cover layers of the LPS core close to the cell surface but not the terminal layers located further out. Agreeing to this notion, RBP binding became noticeably less efficient (decrease of RBP-reporter signal) for the RBP of ΦA1122 phage when capsule formation increased at 37 °C. A likely cause for this could be a shielding effect of the growing F1 antigen capsule against the ΦA1122 phage surface receptor Kdo/Ko in the core of the LPS of *Y. pestis* [18,19]. The Kdo/Ko surface receptors are directly connected to lipid A and are therefore significantly closer to the cell surface than the terminal N-acetylglucosamine, which serves as the likely surface receptor for the RBP of phage L-413C [19]. This may explain why the RBP-reporter of phage L-413C was still able to bind to its cognate, more peripherally positioned, primary surfaces receptor but the RBP of the phage ΦA1122 was not. 

Strains from the *Y. pseudotuberculosis* complex, comprising *Y. pseudotuberculosis*, *Y. pestis*, *Y. similis* and *Y. wautersii*, form a clade of closely related species based on 16S rRNA gene sequences [7] making it necessary to test RBP-reporter specificity on members of this clade. Other relatives of *Y. pestis* tested were strains of the enteropathogenic species *Y. enterocolitica*, which is divided into the subspecies *enterocolitica* and *palearctica* [33]. At a growth temperature of 28 °C, the RBP of both phages L-413C and ΦA1122 showed high specificities for *Y. pestis* clearly discriminating against other species of the *Y. pseudotuberculosis* complex (incl. *Y. pseudotuberculosis*) and *Y. enterocolitica*. Even at 37 °C the RBP tested negative for binding to *Y. similis*, *Y. wautersii* and *Y. enterocolitica*. While no binding of the RBP of phage L-413C to *Y. pseudotuberculosis* occurred when cultures were grown at 37 °C, the RBP of phage ΦA1122 did, albeit weaker than to *Y. pestis* (Figure 5 and Figure 6). This can be expected, for the receptor of phage ΦA1122 is the Kdo/Ko region of the LPS nuclear structure, which occurs in the LPS of both *Y. pestis* and *Y. pseudotuberculosis* but not in the LPS of e.g., *Y. enterocolitica* [34]. Skurnik et al. (2012) demonstrated that phage ΦA1122 does not infect *Y. enterocolitica* strains agreeing with the lack of RBP binding in our specificity tests [34]. This difference in binding to *Y. pseudotuberculosis* cells at 37 °C of phage L-413C- and ΦA1122-RBP also fits to earlier observations when the parental complete phages were used to infect cells in plaque assays. Phage ΦA1122 infected *Y. pseudotuberculosis* at 37 °C as demonstrated by different studies using a large number of tested strains [16,35]. This earlier work also indicated that in practice differentiation between *Y. pestis* and *Y. pseudotuberculosis* by susceptibility to phage ΦA1122 is robust up to 28 °C [16]. The temperature-regulated biosynthesis of the O-PS chains leads to the formation of long O-PS chains below 30 °C, whereas short chains or no O-PS formation occurs at temperatures above 37 °C [34]. Thus, as production of *Y. pseudotuberculosis* O-PS is strongly diminished at temperatures above 30 °C, attachment of phage ΦA1122 becomes possible. Therefore, Filippov et al. (2011) proposed that the O-antigen blocks the phage receptor in the inner core of *Y. pseudotuberculosis* LPS at lower temperatures [19].

Conversely, in the present work binding of L-413C-RBP was exclusive to *Y. pestis* cells. We tested four *Y. pseudotuberculosis* strains and found them to be negative for binding. Tests with more than 1200 strains of *Y. pseudotuberculosis* and 7000 strains of *Y. pestis* (isolated from various plaque foci around the world) also confirmed that phage L-413C has a uniquely high specificity for *Y. pestis* [36]. According to that publication, there are only 10 atypical *Y. pestis* strains resistant to phage L-413C. None of these strains were available for the present work.

In an earlier study, specificities of phages against *Y. pestis* were evaluated. For instance, phages of so-called serovar 1 usually exhibit only partial specificity and lyse between 74 and 100% of the tested *Y. pestis* strains, a significant fraction of *Y. pseudotuberculosis* (25–65%), *Y. enterocolitica* (up to 15%) and other enterobacterial isolates [16]. Phage ΦA1122 scores top specificity values within this group [35]. The phage was capable of infecting some *Y. pseudotuberculosis* strains at 37 °C but after cultivation at 20 °C exclusively lysed *Y. pestis*. These observations were confirmed by further testing; yet two *Y. pestis* strains among the thousands deposited in the culture collection of the Center for Disease Control and Prevention (CDC) proved to be resistant to this phage [16]. The authors proposed that if *Y. pestis* isolates from plague outbreaks in the former Soviet Union and Mongolia, which included some atypical strains as well as some naturally phage-resistant strains, were included, the number of resistant strains would increase. Nevertheless, due to its specific and broad strain infectivity, the ΦA1122 phage has been in use by the CDC, WHO and the US Army Medical Research Institute for Infectious Diseases as a diagnostic standard (as phage lysis assay) for the confirmatory identification of *Y. pestis* [37,38]. Phage ΦA1122 has later been developed into a recombinant, bioluminescence-mediating reporter phage facilitating detection of *Y. pestis* at an limit of detection of 10^2^ cells within 60 min with little off-target signals from *Y. pseudotuberculosis* or *Y. enterocolitica* [14].

Phage L-413C belongs to a different serovar group (serovar 2) than phage ΦA1122. Typically, phages of this group can multiply in a wide range of *Yersinia* spp. and, with a few exceptions, such as phage L-413C, have low specificities [15]. These authors suspected that phage L-413C was highly specific for *Y. pestis* because the phage lysed all *Y. pestis* strains tested but failed to successfully infect other *Yersinia* spp. and the majority of other enterobacteria. So far, only a few “restriction deficient” *E. coli* C-strains have been identified as hosts of phage L-413C [15].

While providing specific identification and requiring little monetary investments, phage plaque assay testing is time consuming. More ideally, rapid and specific confirmatory detection techniques are needed for unambiguous pathogen identification. For *Y. pestis*, lateral flow immunochromatographic assays employing F1 capsule antibodies have been in successful use for many years and new derivatives are still being developed [10,31,39]. For instance, such invaluable rapid tests have been instrumental for on-site plague diagnostics in Madagascar since 2002 [40]. Earlier it was shown that the rapid F1 capsule test is more efficacious than bacteriological methods or the F1 ELISA as it correctly identified significantly more positive clinical samples [41]. Using a combination of bacteriological methods and F1 ELISA as reference standard, the positive predictive value of the rapid test was 90.6% and the negative predictive value 86.7% [41]. In the work at hand, with a more modest sample size, RBP-reporters derived from phages L-413C or ΦA1122 all 7 out of 7 tested *Y. pestis* strains were all positive, all 10 near neighbors were identified as negative, giving a specificity of 100% at 28 °C. However, at 37 °C, due to the expected detection of *Y. pseudotuberculosis* strains at this temperature, these values dropped to 60% (6/10) for phage ΦA1122. Though the work described herein is a proof-of-principle study, in the future testing of larger panels of known positive and negative isolates will be required for full validation. Atypical *Y. pestis* strains [36] should also be included, which are currently not available to us.

## 4. Conclusions

In conclusion, we have tested a hypothesis formulated earlier that the gene products of the genes *gpH* (phage L-413C) and *gp17* (phage ΦA1122) are the likely RBPs of these highly specific phages of *Y. pestis*. By constructing fusions of phage RBPs and fluorescent proteins, we developed an assay for the rapid detection of *Y. pestis* cells which is species specific at 28 °C for both RBP tested and remains specific at 37 °C when the reporter included the RBP from phage L-413C. Use of the RBP from phage L-413C is recommended also because this RBP-reporter seems to be able to even label encapsulated cells grown at 37 °C found after infection of mammal hosts. Such diagnostic testing is facilitated by the finding that RBP-reporter binding can also occur on formaldehyde-inactivated *Y. pestis* specimens.

It is therefore our expectation that such RBP-reporter assays can be applied after validation for the testing of mixed bacterial cultures (e.g., from collected fleas) and of clinical matrices, such serum or environmental samples for stationary diagnostics or field laboratories.

## 5. Materials and Methods 

### 5.1. Strains, Phages and Plasmids

Strains, phages and plasmids used in this work are listed in Supplementary Table S1.

### 5.2. Media, Buffer, Additives and Growth Conditions Including Growth Curves

*E. coli* cultures were grown in LB broth or on LB-agar (Merck KGaA, Darmstadt, Germany) containing, if required, 20 μg/mL of gentamycin and 100 µg/mL carbenicillin (Carl Roth, Karlsruhe, Germany). *Y. pestis* and other *Yersinia* spp. were cultivated at 28 °C (or at 6, 20 or 37 °C) on brain-heart infusion agar plates (BHI, Merck KGaA, Darmstadt, Germany) or in 250 mL baffled flasks containing 50 mL BHI broth (TSB, Merck KGaA, Darmstadt, Germany) with shaking at 120 rpm. *Y. pestis* strains of risk group 3 (RG-3) were cultivated in the biosafety level 3 (BSL-3) laboratory at the Bundeswehr Institute of Microbiology (IMB). Before subjected to further testing, cells of RG-3 strains were chemically inactivated with 70% (v/v) ethanol unless stated otherwise. *Yersinia* phages were propagated on *Y. pestis* EV76 cultures by standard protocols as described previously [42].

### 5.3. Cloning of Phage RBP Gene Fusions

PCR primer oligonucleotides were designed using Geneious Prime software (Biomatters Limited, Auckland, New Zealand) from DNA sequences of phage ΦA1122 (accession #NC_004777), phage L-413C (accession #NC_004745), mCherry-pBAD or eGFP-pBAD (Supplementary Table S2). Cloning of phage RBP genes was carried out as described previously [43]. For cloning of genes of fluorescent proteins and the putative RBP genes, the primers were designed in such a way that the recognition sites for SalI, EcoRI and BsrGI upstream and XhoI, PstI and BsiWI downstream of the fluorescence genes, which were inserted into the vector chassis pASG-IBA 105 (IBA GmbH, Göttingen, Germany), can be used for subsequent cloning of additional genes. Digesting these chassis vectors with XhoI and BsiWI and subsequent ligation of the putative RBP genes enabled cloning of RBP-reporters. In the respective expression vectors (Supplementary Table S1), the N-terminal end of the encoded recombinant protein carries a Twin-Strep tag [44] and the putative head domains of the RBP are located at the C-terminus. To this end, genes of fluorescent proteins eGFP (green) or mCherry (red) were PCR-amplified from mCherry-pBAD or pEGFP-C1, respectively using primer pairs mCherry forward/reverse and eGFP forward/reverse (Supplementary Table S2) creating overhangs containing restriction sites for Esp3I for cloning into pASG-IBA105 expression plasmid downstream of the *Twin-Strep-tag* nucleotide sequence. One-step Esp3I digestion and ligation was carried out using StarGate Direct transfer cloning System (IBA GmbH, Göttingen, Germany) as described in the manufacturer’s protocol and plasmids were transformed into NEB Turbo cells (New England Biolabs GmbH, Frankfurt am Main, Germany). Clones were confirmed by Sanger sequencing (Eurofins Genomics Germany GmbH, Ebersberg, Germany) of their recombinant pASG-mCherry/pASG-eGFP plasmids using primers flanking the insert.

Genes encoding RBP were PCR-amplified from custom-synthesized GeneBlocks (ThermoFisher Scientific, Darmstadt, Germany) of *E. coli* codon-optimized *gpH* (phage L-413C, accession #NC_004745) or *gp17* (phage ΦA1122, accession #NC_004777), respectively using primer pairs L-413Cp19-RBP F/R and A1122p42-RBP F/R (Supplementary Table S2). PCR samples were analyzed by agarose gel-electrophoresis and cut with XhoI and BsiWI (New England Biolabs GmbH, Frankfurt am Main, Germany) to generate cohesive (sticky) ends, ligated into expression vectors (Supplementary Table S1) cut with the same restriction endonucleases and transformed into chemically competent *E. coli* NEB Turbo cells (New England Biolabs GmbH, Frankfurt am Main, Germany) [42]. Individual clones were grown, inserts confirmed by colony-PCR and plasmids isolated using the QIAprep Spin Miniprep Kit (QIAGEN, Hilden, Germany). DNA sequencing of recombinant plasmids was performed by commercial services (Eurofins Genomics, Ebersberg, Germany).

### 5.4. Heterologous Production of Fluorescent RBP-Reporter Fusion Proteins

DNA of expression plasmids was transformed into chemically competent *E. coli* ArcticExpress cells. A single colony used to inoculate an overnight culture which was diluted 1% into fresh LB medium and incubated with shaking (120 rpm) at 37 °C. After derepression of heterologous gene expression by adding anhydrotetracycline (AHT; IBA GmbH, Göttingen, Germany) at an optical density at 600 nm (OD600) of 0.4–0.8 [45], incubation was continued for 24 h at 12 °C. Successful protein biosynthesis was monitored by a change in the color of the culture medium by fluorescing *E. coli* cells. Cells were harvested by centrifugation (7500× *g*, 10 min) and cell pellets stored at −20 °C until further use. Cell pellets were resuspended and processed as described previously [43]. Cell lysis was accomplished by mechanical means using a French Press (Emulsiflex-C3; Avestin Europe GmbH, Mannheim, Germany). Affinity chromatography was conducted making use of the Twin-Strep-tag-Streptactin^®^XT system (IBA GmbH, Göttingen, Germany) including centrifugation columns (Strep-Tactin^®^XT Spin Columns, IBA GmbH, Göttingen, Germany), gravity-flow columns (Strep-Tactin^®^XT sepharose, IBA GmbH, Göttingen, Germany) or Fast protein liquid chromatography (FPLC, Äkta pure, GE Healthcare Life Science, Munich, Germany) according to the manufacturer’s protocols. Proteins were analyzed for correct sizes and purity by denaturing sodium dodecyl-sulfate polyacrylamide gel electrophoresis (SDS-PAGE) stained with PierceTM Reversible Protein Protein Stain (ThermoFisher Scientific, Darmstadt, Germany) and Strep-tagged protein detection after Western blotting onto nitrocellulose membranes using horseradish peroxidase-coupled StrepMAB antibodies (IBA GmbH, Göttingen, Germany) with enhanced chemiluminescence (ECL) solution (ClarityTM Western ECL Substrat Kit, Bio-Rad Laboratories, Munich, Germany) as chromogenic substrate.

### 5.5. Inactivation of *Y. pestis* Cells

For inactivation of *Y. pestis* cells with 70% ethanol, 1–2 mL of culture were centrifuged at 5000× *g* for 2 min at room temperature (RT), the pellet resuspended in 300 µL phosphate-buffered saline (PBS, pH 7.2), 1200 µL of 100% ethanol added and the mixture incubated for at least 15 min with mixing by frequently inverting the tube at RT. Cells were pelleted at 5000× *g* for 2 min at RT and the supernatant removed. Cells were washed with 1.5 mL of PBS and resuspended in 1 mL of fresh PBS. For paraformaldehyde (PFA) inactivation, 1–2 mL of *Y. pestis* cultures were centrifuged at 5000× *g* for 2 min at RT and the pellet resuspended in 1 mL PBS. After another wash step with PBS the cell pellet was resuspended in 250 µL fresh PBS, 750 µL ice-cold, 4% PFA solution was added and the mix incubated at RT for 1 h. The sample was then centrifuged at 5000× *g* for 2 min at RT and the pellet resuspended and washed by centrifugation with PBS and finally resuspended in 1 mL fresh PBS. For heat inactivation, 200 µL aliquots of *Y. pestis* cultures were washed in PBS by centrifugation, resuspended in 200 µL of PBS and incubated for 30 min at 95 °C in a heating block with temperature-controlled lid. Inactivation by peracetic acid (Terralin PAA, Schülke & Mayr GmbH, Norderstedt, Germany) was performed by mixing 750 µL aliquots of bacterial cultures with 750 µL of 4% Terralin PAA followed by 15 min incubation at RT. Cells were washed twice with 1.5 mL of PBS and resuspended in 1 mL of fresh PBS. Inactivated cells were stored at 4 °C until further use.

### 5.6. Labeling of *Y. pestis* and Other Bacterial Cells with RBP-Reporters or Antibodies

RBP-reporter assays were carried out as described previously for *B. anthracis* with slight modifications [43]. Briefly, 500 µL of an overnight culture of *Y. pestis* or other *Yersinia* spp. strains was used to inoculate 50 mL of fresh BHI medium in a 250 mL baffled shaking flask and the culture was incubated at 6, 20, 28 or 37 °C with shaking at 100 rpm. At different time points, OD_600_ was measured and a sample taken equivalent to 100 µL of an OD_600_ of 1. When cultures were grown on solid media, agar plates were used. Single colonies were picked, resuspended in 100 µL PBS and diluted to a final OD_600_ of 1. Samples were pelleted by centrifugation at 5000× *g* for 5 min at RT in 1.5 mL centrifugation tubes, resuspended in PBS, mixed with 3 µg of purified RBP-reporter protein and incubated for 10 min at room temperature. When testing for capsule formation 0.3 µg of F1-antigen antibody (*Y. pestis* F1-Antigen Antibody [YPF19], GeneTex Inc., Irvine, CA, USA) was added alongside the RBP-reporter and incubation time was increased to 1 h. After a washing step using PBS (5000× *g* for 2 min), 0.3 µg of goat anti-mouse Alexa Fluor^®^ 647 antibody (if RBP of phage ΦA1122 was used) or antibody Alexa Fluor^®^ 488 (if RBP of phage L-413C was used, ThermoFisher Scientific, Darmstadt, Germany) was added, followed by incubation at room temperature for 1 h. After a final washing step with PBS (5000× *g* for 2 min) 4 µL were transferred into a well of a chamber slide with lid (µ-slide 8 Well, Ibidi GmbH, Martinsried, Germany). For proper microscopic analysis of cells, suspensions were covered with a 1 mm thick agar-agar pad serving as a coverslip (1% molten agar-agar solidified between two microscopy slides). Samples were analyzed for cells emitting mCherry (excitation: 587 nm, emission: 610 nm) or eGFP signal (excitation: 488 nm, emission: 507 nm) as well as for signal emanating from secondary antibodies Alexa Fluor^®^ 488 (excitation: 490 nm, emission: 525 nm) and Alexa Fluor^®^ 647 (excitation: 650 nm, emission: 665 nm) using Axio Observer Z1 700 Confocal Laser Scanning Microscope (Carl Zeiss, Oberkochen, Germany).

## Supplementary Materials

The following are available online at https://www.mdpi.com/2076-0817/9/8/611/s1, Table S1: Strains, phages and plasmids used in this work; Table S2: Oligonucleotide primers; Figure S1: Western blot of heterologously produced RBP fusion reporter proteins; Figure S2: Binding of RBP-reporters to growing cultures of *Y. pestis* EV76 cells at 28 °C.
