# Supplementary material for: Specific Detection of Yersinia pestis Based on Receptor Binding Proteins of Phages
Source: Pathogens. 2020 Jul 27;9(8):611. doi: 10.3390/pathogens9080611 (PMC7460101; doi:10.3390/pathogens9080611)
Supplement: Supplementary file 1 [file pathogens-09-00611-s001.pdf]

Supplementary material for: **Specific detection of *Yersinia pestis* based on receptor binding proteins of phages**

Friederike Born ‡, Peter Braun ‡, Holger C. Scholz , and Gregor Grass *

Bundeswehr Institute of Microbiology (IMB), Dept. of Bacteriology and Toxinology, 80937 Munich, Germany; friederikeleonieborn @bundeswehr.org (F.B.); peter3braun@bundeswehr.org (P.B.); holger1scholz@bundeswehr.org (H.C.S.)

* Correspondence: gregorgrass@bundeswehr.org; Tel.: +49-99-2692-3981

† Both authors contributed equally to this work.

Supplementary Table S1: Strains, phages and plasmids used in this work.

Strain/phage/plasmid	Characteristics	Reference
E. coli BL21 (DE3)	*F⁻ ompT hsdSB* ($r_B^- m_B^-$) *gal dcm* (DE3)	ThermoFisher Scientific, Darmstadt, Germany
E. coli ArcticExpress™ (DE3)	*E. coli B F⁻ ompT hsdS*(r_B^- m_B^-) *dcm*$^+$ *Tet*^{r} *gal λ* (DE3) *endA Hte [cpn10cpn60 Gent*^{r}*]*	Agilent Technologies, Waldbronn, Germany
E. coli NEB Turbo	*F´ proA*$^+$*B*$^+$ *lacI*^{q} *ΔlacZ M15/ fhuA2 Δ(lac-proAB) glnV gal R(zgb-210::Tn10)Tet*^{S} *endA1 thi-1 Δ(hsdS-mcrB)*	New England Biolabs GmbH, Frankfurt am Main, Germany
Yersinia pestis EV 76	Attenuated deletion mutant (*pgm/hms*$^-$), vaccine strain	[1]
Yersinia pestis Kuma	Bv. Antiqua	[2]
Yersinia pestis M23	Bv. Orientalis	[3]
Yersinia pestis TS	Bv. Orientalis	[4]
Yersinia pestis G8786	Bv. Pestoides, isolated from Georgia	[5]
Yersinia pestis Rodent 24	Bv. Medievalis, isolated from rodent in Kurdistan	[6]
Yersinia pestis NCTC 10029	Bv. Antiqua, isolated from human, Nairobi, Kenya, 1958	The National Collection of Type Cultures (NCTC) for bacteria, UK
Yersinia pseudotuberculosis (B-1706)	Isolated from beaver (Germany)	Strain collection of the Bundeswehr Institute of Microbiology
Yersinia pseudotuberculosis (Y-714)	O:1a	Strain collection of the Bundeswehr Institute of Microbiology
Yersinia pseudotuberculosis (Y-715)	O:1a	Strain collection of the Bundeswehr Institute of Microbiology

Strain/phage/plasmid	Characteristics	Reference
Yersinia pseudotuberculosis (Y-716)	O:1a	Strain collection of the Bundeswehr Institute of Microbiology
Yersinia similis Y228^T (DSM18211)	O:6, isolated from rabbit	[7]
Yersinia wautersii (Y-428) (*Y. pseudotuberculosis* ('Korean group')	O:4a, isolated from badger	[8]
Yersinia enterocolitica (Y-929)	O:3, isolated from hare	Strain collection of the Bundeswehr Institute of Microbiology
Y. enterocolitica subsp. *palearctica* (DSM 13030)	O:3, human isolate	DSMZ-German Collection of Microorganisms and Cell Cultures
Phage L-413C (NC_004745)	Caudovirales; Myoviridae; Peduovirinae; Peduovirus: Morphotyp A1 - Serovar 2	[9]
Phage ΦA1122 (NC_004777)	Caudovirales; Podoviridae; Autographivirinae; Teseptimavirus; Morphotyp C1 - Serovar 1	[10]
pEGFP-C1	eGFP-Template	Takara Bio Europe SAS (Saint-Germain-en-Laye, France)-
mCherry-pBAD	mCherry-Template	[11]; Addgene plasmid #54630; http://n2t.net/addgene:54630
pASG-IBA 105*	*tet*-Promoter, *Twin-Strep-tag*, *lacP/Z*, *f1* ori, Amp^R, tetR, *colEI* ori,	IBA GmbH, Göttingen, Germany
pASG 105::TST::eGFP:: L-413C-RPB	Derivative of pASG-IBA 105, encodes fusion protein of Twin-Strep-tag, eGFP, gpH (RBP)	This work
pASG 105::TST::mCherry::L-413C-RPB	Derivative of pASG-IBA 105, encodes fusion protein of Twin-Strep-tag, mCherry, GpH (RBP)	This work
pASG 105::TST::eGFP:: ΦA1122-RPB	Derivative of pASG-IBA 105, encodes fusion protein of Twin-Strep-tag, eGFP, Gp17 (RBP)	This work
pASG 105::TST::mCherry:: ΦA1122-RPB	Derivative of pASG-IBA 105, encodes fusion protein of Twin-Strep-tag, mCherry, Gp17 (RBP)	This work

Supplementary Table S2: Oligonucleotide primer sequences used in this work.

**Oligonucleotide**	**Sequence (5'-3')**
L-413Cp19-RBP F	AAA CTC GAG TCT ACC AAA TTC AAA ACC GTT ATC ACC
L-413C-p19RBP R	AAA CGT ACG AAC CTG AGC AAC GTT GTA CCA

A1122p42-RBP F	AAA CTC GAG GCT AAC GTT ATC AAA ACC GTT CTG A
A1122p42-RBP R	AAA CGT ACG AAC ATC TTC AAC AGC GAT AG
eGFP forward	AGCGCGTCTCCAATGGTCGACGGTGAATTCGGCTGTACAGTGAGCAAGGGCGAGGAGCTGTTCAC
eGFP reverse	AGCGCGTCTCCTCCCCGTACGGCCCTGCAGACCCTCGAGCTTGTAGAGCTCGTCCATGCCGAGAG
mCherry forward	AGCGCGTCTCCAATGGTCGACGGTGAATTCGGCTGTACAGTTAGTAAAGGAGAAGAAAATAACATGGC
mCherry reverse	AGCGCGTCTCCTCCCCGTACGGCCCTGCAGACCCTCGAGTTTGTATAGTTCATCCATGCCACCAG

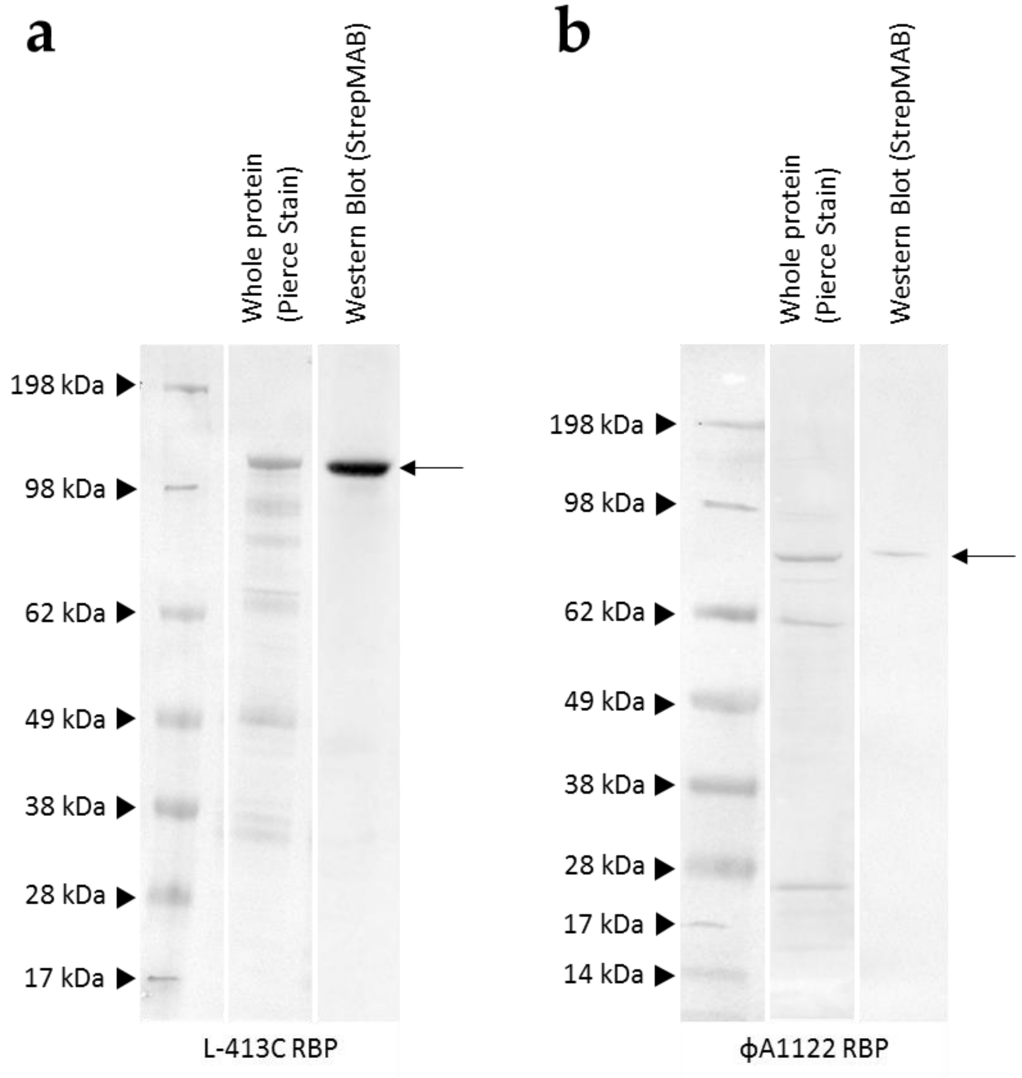

Supplementary Figure S1: Western blot of heterologously produced RBP fusion reporter proteins. Affinity purified proteins were subjected to SDS-PAGE, stained (Pierce stain) after transfer onto a nitrocellulose membrane and the TST epitope detected using a HRP-conjugated TST-antibody (StrepMAB, IBA GmbH, Göttingen, Germany). Expected sizes of RBP eGFP reporters: (a) L-413C RBP 130 kDa and (b) ΦA1122 RBP 93,5 kDa (indicated by arrows). Letters indicate size-positions of the protein size marker (SeeBlue Plus2 prestained, ThermoFisher Scientific, Darmstadt, Germany).

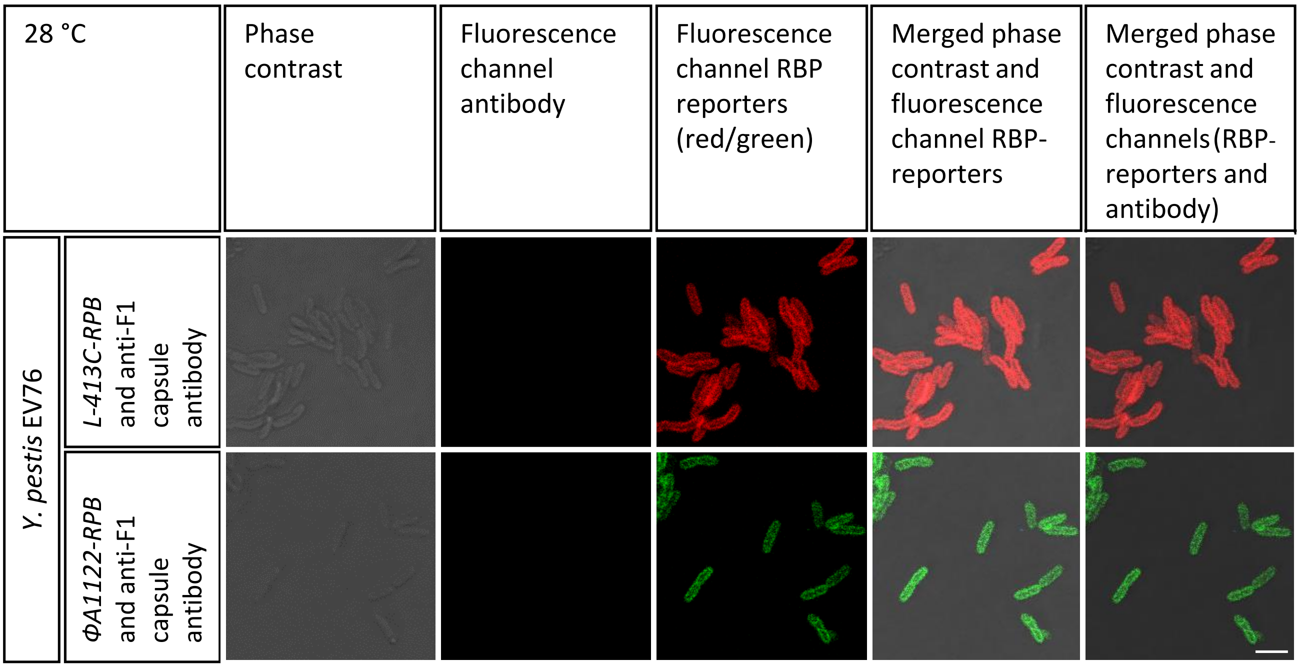

Supplementary Figure S2: Binding of RBP-reporters to growing cultures of *Y. pestis* EV76 cells at 28°C. In this representation, capsule formation was checked by incubation with a monoclonal anti-F1 capsule antigen antibody in combination with secondary antibody labelled with Alexa Fluor 488 for co-detection with L-413C-RBP-reporters or Alexa Fluor 647 for co-detection with ΦA1122-RPB-reporter, respectively. RBP binding to *Y. pestis* EV76 cells 6 h after inoculation at 28 °C is shown as individual representative micrographs for phage L-413C mCherry-RBP-reporter (red signals) or phage ΦA1122 eGFP-RBP-reporter (green signals) (as separate phase contrast, separate fluorescence or merged channels as indicated) (scale bar: 5 μm).

References

1. Saltykova RA, Faĭbich MM [Experience from a 30-year study of the stability of the properties of the plague vaccine strain EV in the USSR]. *Zh Mikrobiol Epidemiol Immunobiol* **1975**: 3–8.
2. Paoli GC, Sommers CH, Scullen OJ, Wijey C Inactivation of avirulent *pgm*(+) and Δ*pgm Yersinia pestis* by ultraviolet light (UV-C). *Food Microbiol* **2014**, *44*: 168–172.
3. Fetherston JD, Schuetze P, Perry RD Loss of the pigmentation phenotype in *Yersinia pestis* is due to the spontaneous deletion of 102 kb of chromosomal DNA which is flanked by a repetitive element. *Mol Microbiol* **1992**, *6*: 2693–2704.
4. Ginoza HS, Matney TS Transmission of a resistance transfer factor from *Eschierichia coli* to 2 species of *Pasteurella*. *J Bacteriol* **1963**, *85*: 1177–1178.
5. Golubov A, Neubauer H, Nölting C, Heesemann J, Rakin A Structural organization of the pFra virulence-associated plasmid of rhamnose-positive *Yersinia pestis*. *Infect Immun* **2004**, *72*: 5613–5621.
6. Baltazard M, Bamanyar M, Mofidi C, Seydien B [Le foyer de peste du Kurdistan] The Kurdistan plague focus. *Bull World Health Organization* **1952**, *5*: 441–472.
7. Sprague LD, Scholz HC, Amann S, Busse HJ, Neubauer H *Yersinia similis* sp. nov. *Int J Syst Evol Microbiol* **2008**, *58*: 952–958.
8. Savin C, Martin L, Bouchier C, Filali S, Chenau J, Zhou Z, Becher F, Fukushima H, Thomson NR, Scholz HC, Carniel E The *Yersinia pseudotuberculosis* complex: characterization and delineation of a new species, *Yersinia wautersii*. *Int J Med Microbiol* **2014**, *304*: 452–463.

9. Garcia E, Chain P, Elliott JM, Bobrov AG, Motin VL, Kirillina O, Lao V, Calendar R, Filippov AA Molecular characterization of L-413C, a P2-related plague diagnostic bacteriophage. *Virology* **2008**, *372*: 85–96.
10. Garcia E, Elliott JM, Ramanculov E, Chain PS, Chu MC, Molineux IJ The genome sequence of *Yersinia pestis* bacteriophage phiA1122 reveals an intimate history with the coliphage T3 and T7 genomes. *J Bacteriol* **2003**, *185*: 5248–5262.
11. Shaner NC, Campbell RE, Steinbach PA, Giepmans BN, Palmer AE, Tsien RY Improved monomeric red, orange and yellow fluorescent proteins derived from *Discosoma* sp. red fluorescent protein. *Nat Biotechnol* **2004**, *22*: 1567–1572.
